# Prognostic value of left atrial strain in significant aortic valve disease: a systematic review and meta-analysis

**DOI:** 10.3389/fcvm.2025.1667871

**Published:** 2025-09-16

**Authors:** Na Chen, Wenhui Gu, Jun Wu

**Affiliations:** 1 Department of Echocardiography, The Second Affiliated Hospital of Dalian Medical University, Dalian, China; 2 Department of Echocardiography, The First Affiliated Hospital of Dalian Medical University, Dalian, China

**Keywords:** aortic stenosis, aortic regurgitation, left atrial strain, peak left atrial longitudinal strain, systematic review, meta-analysis

## Abstract

**Background:**

Previous studies on aortic valve disease have mainly focused on the left ventricle, but increasing evidence suggests that left atrial strain also has prognostic value in significant aortic valve disease.

**Objective:**

To systematically evaluate the prognostic value of left atrial strain in significant aortic valve disease.

**Methods:**

Multiple electronic databases were searched for studies evaluating significant aortic stenosis (AS) or aortic regurgitation (AR) using peak left atrial longitudinal strain (PALS) from the inception of each database to 1 February 2025. There were no language or regional restrictions. The primary endpoint was a composite outcome comprising all-cause mortality, hospitalization for heart failure, aortic valve replacement, pulmonary hypertension, and postoperative new-onset atrial fibrillation.

**Results:**

A total of 25 studies were included, involving 7,195 patients, with 2,039 (28%) patients experiencing primary endpoint events. The PALS was lower in the positive group (EVENT+) compared to the negative group (EVENT−) (SMD = −1.03, 95% CI [−1.22, −0.84], *p* < 0.05). For each unit increase in PALS, the risk of the primary endpoint event decreased by 7% (HR = 0.93, 95% CI [0.91, 0.96], *p* < 0.001). PALS exhibited consistent incremental predictive value in both the AR and AS cohorts, although the strength of its effect and the underlying mechanisms varied between groups.

**Conclusion:**

PALS is an independent predictor of adverse cardiovascular events in patients with significant aortic valve disease. PALS has certain value in the prognosis of significant aortic valve disease.

**Systematic Review Registration:**

[www.crd.york.ac.uk/prospero/], identifier [CRD 42024623883].

## Introduction

According to multiple studies, aortic valve disease accounts for 61% of all deaths from valvular heart disease. Aortic valve disease includes conditions such as aortic stenosis (AS) and aortic regurgitation (AR), which are closely associated with aging and chronic cardiovascular disease. Among these, aortic stenosis (AS) is the most prevalent valvular heart disease in developed countries, currently affecting approximately 9 million individuals worldwide, and its incidence continues to rise in parallel with population aging and the growing burden of atherosclerosis. In 2019, AS caused approximately 127,000 deaths worldwide, with related losses amounting to 1.8 million disability-adjusted life years. AR is associated with diastolic rather than systolic hypertension, and its incidence has also increased in developed countries (1). Previous studies on significant valvular heart disease have primarily focused on the left ventricle (LV), with relatively little research conducted on the left atrium (LA). However, LA is a bridge connecting the systemic circulation and pulmonary circulation, closely related to cardiopulmonary function and affecting the patient's symptoms. Moreover, in recent years, an increasing number of studies have suggested that left atrial strain (LAS), particularly peak atrial longitudinal strain (PALS), has important prognostic value in significant aortic valve disease (2). However, studies on the prognostic value of PALS in aortic valve disease, particularly in AR, are mostly single-center, with small sample sizes and controversial results. Currently, there is a lack of meta-analyses on the prognostic value of PALS in significant aortic valve disease. Therefore, this study aims to explore the prognostic value of PALS in significant aortic valve disease through systematic evaluation and meta-analysis.

## Methods

### Search strategy and study inclusion

This study was conducted in strict accordance with the Preferred Reporting Items for Systematic Reviews and Meta-Analyses (PRISMA) guidelines. The protocol was registered with the International Prospective Register of Systematic Reviews (PROSPERO) (CRD 42024623883).

A systematic search in PubMed, Cochrane, Web of Science, Embase, Ovid, and CNKI databases was performed between their inception to 1 February 2025. Key words including: atrial function, atrial deformation, atrial longitudinal strain, aortic stenosis, aorta insufficiency, aorta regurgitation and aortic incompetence. Additionally, the reference lists of all included articles were hand-searched to identify additional eligible studies, and no language restrictions were imposed during the entire search process. The specific search strategies for each database are detailed in Supplementary Table S1. Two investigators independently performed the two-stage screening process: first, titles and abstracts were screened, and then full-text articles were reviewed for eligibility (NC and JW). Disagreements were resolved by a third author (WHG) to reach a consensus.

Inclusion criteria: (1) The study included patients aged ≥18 years diagnosed with moderate-to-severe or severe AS or AR by echocardiography. (2) PALS was measured using speckle tracking echocardiography (STE). (3) Prospective or retrospective studies in which the composite endpoint was defined as all-cause mortality, hospitalization for heart disease, aortic valve replacement, pulmonary hypertension, and postoperative new-onset atrial fibrillation (AF).

Exclusion criteria: (1) Studies that employed cardiac magnetic resonance imaging for PALS quantification were excluded to avoid heterogeneity arising from methodological disparities. (2) Case reports, reviews, letters, and editorials were also excluded. (3) In instances where overlapping datasets yielded multiple publications, only the most recent or methodologically complete report was retained.

### Data extraction

The two authors (NC and JW) independently extracted the data and summarized them in a data extraction file. Any discrepancies were resolved through consensus or consultation with the third author (WHG). For missing data in eligible studies, researchers attempted to obtain it by contacting the original article authors via email. The quality of the selected trials was evaluated using the Cochrane risk of bias assessment tool from seven aspects.

### Statistical analysis

The data of continuous variables were pooled to calculate the standardized mean difference (SMD) and 95% confidence interval (CI), while the data of binary variables were pooled to calculate the hazard ratio (HR) and 95% CI, to assess their prognostic value for the primary endpoint. The *I*^2^ statistic was used to evaluate statistical heterogeneity among studies. If significant heterogeneity was detected (e.g., *I*^2^ > 50%), a random—effects model was used to construct a forest plot to display the overall effect. A subgroup analysis was conducted to identify the effect of PALS on the prognosis of patients with severe AS and severe AR. Incremental prognostic value of PALS beyond traditional risk models was assessed using incremental discrimination analysis (C-statistic improvement) and the NRI. Sensitivity analyses were performed to exclude the effects of small-sample studies and different subgroup conditions on the overall pooled estimates. Funnel plots were used to assess publication bias. Statistical analysis was performed using Review Manager 5.3 (Cochrane Collaboration, Oxford), and a *p*-value <0.05 was considered statistically significant.

## Results

### Study inclusion

**Figure 1:**
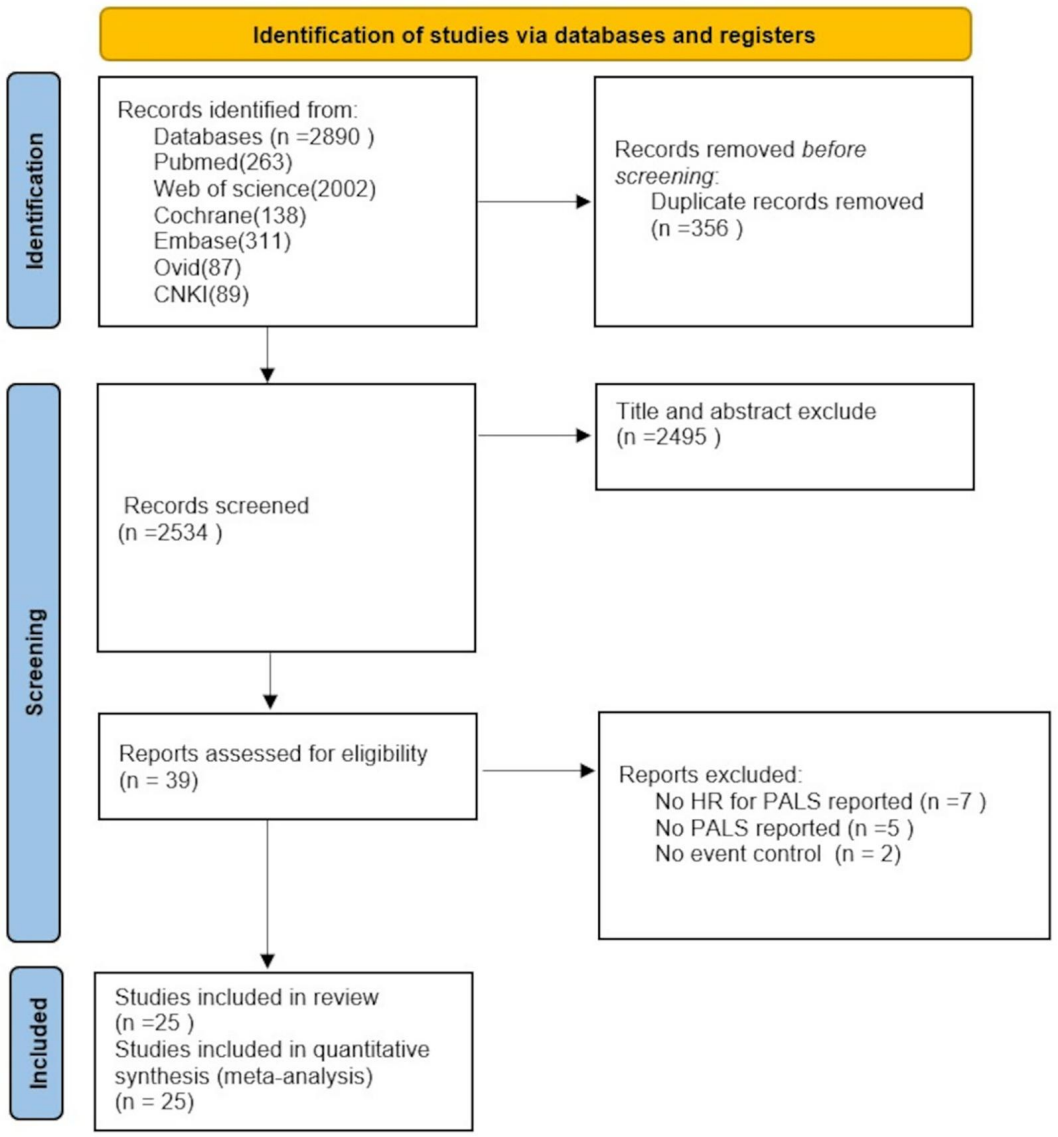
Flowchart of PRISMA for literature screening.

A total of 2,890 records were retrieved according to the search strategy, and 2,534 trials remained after excluding duplicates. Thirty-nine trials remained after initial screening of titles and abstracts. Seven articles that did not report information such as HR values, 5 articles that lacked reporting of PALS, and 2 non-positive and negative event-controlled studies were excluded after detailed reading of the full text. This resulted in the inclusion of 25 studies with a total of 7,195 patients (2–26). The screening process and results are shown in Figure 1.

### Characteristics of the study population

Table 1 summarizes the characteristics of the 25 studies included in the systematic evaluation. All studies were published between 2014 and 2025. Among them, 11 studies were single-center or multicenter prospective cohort studies. Regarding the patient population, 4 studies reported only AR, 20 studies reported only AS, and 1 study reported both AS and AR. The mean age range of patients was 49–82 years, and 56% were male. Of the 11 studies reporting comorbidities, the most common diseases were hypertension (68.1%) and diabetes (27.5%). Among the reported studies, mean baseline PALS ranged from 12.4 ± 6.9% to 39.4 ± 17.4%. The composite endpoints of all-cause death, cardiac hospitalization, postoperative new-onset AF, and pulmonary hypertension were seen in 2013 (27.5%) patients during follow-up from 0.1 to 57 months.

**Table 1:** Study characteristics.

Publication	Design	Sample size	Group	Age (years)	Male (%)	PALS (%)	Primary endpoint	Follow-up, month	Events, *n* (%)
Pessoa-Amorim et al. (13)	Prosp	114	AS	74 ± 8.6	76 (67)	25.5 ± 10.9	New onset of AF	0.10 (0.03–0.13)	36 (32)
Anastasius et al. (17)	Prosp	109	AS	81 ± 7.3	53 (49)	18 ± 14	HF hospitalization, ACD	12	8 (7.3)
Benfari et al. (25)	Prosp	67	AS	72 ± 8	23 (34)	23 ± 7	ACD, HF hospitalization, ischemic disease or AF onset	26.30 ± 8.20	8 (12)
Butcher et al. (2)	Retro	601	AS	81 (76–85)	318 (53)	NR	ACD	40 (26–58)	258 (43)
Cameli et al. (24)	Prosp	76	AS	66.5 ± 12.1	42 (69)	33.6 ± 9.5	POAF	0.06 (0.03–0.10)	15 (19.7)
				71.5 ± 10.1	10 (67)	22.5 ± 7.1			
Parasca et al. (15)	Prosp	132	AS	76.6 ± 7.5	56 (42)	12.4 ± 6.9	ACD and rehospitalization	29.60 (4.50–36.50)	38 (28.7)
Galli et al. (23)	Retro	128	AS	78.9 ± 9.1	73 (57)	18.4 ± 7.9	HF, cardiac-related hospitalizations, ACD	14 (9–20)	38 (30)
Imanishi et al. (21)	Retro	40	AS	73 ± 9	13 (65)	18.7 ± 3.0	HF Symptoms	0	20 (50)
				78 ± 6	5 (25)	13.0 ± 2.3			
Jenner et al. (20)	Prosp	85	AR	54 (46–63)	56 (86)	26.3 ± 6.7	Diastolic dysfunction grade ≥ 2, LVEF < 50%, or LVEDVI above the gender-specific normal range	12	27 (41.5)
			Controls	59 (49–68)	11 (55)	25.7 ± 6.0			0
Lai et al. (19)	Retro	352	AR	59 ± 17	284 (80)	39.4 ± 17.4	ACD	56.40 (21.60–108)	68 (19.3)
Lee et al. (18)	Retro	712	AS	78 ± 12	337 (47)	24.4 ± 13.4	ACD, MACE	18 (12.36–26.50)	93 (13)
Martín et al. (22)	Retro	126	AR	70.1 ± 17.2	75 (59.5)	34.0 ± 12.7	HF hospitalization, cardiovascular mortality, AVR	34.10 (16.50–48.10)	25 (19.8)
Meimoun et al. (16)	Prosp	117	AS	77 ± 10	51 (50)	20 ± 8	HF hospitalization, ACD	25	53 (52)
			Controls	74 ± 6	8 (53)	32 ± 3			0
Salas-Pacheco et al. (11)	Cross-sectional	72	AS&AR	55.1 ± 17.6	41 (56.9)	26.6 (17.7–35.3)	Pulmonary hypertension	NR	34 (47)
Pernigo et al. (14)	Prosp	60	AS	69.3 ± 8.1	50	14.8 ± 2.8	Postoperative AF	0.17	26 (43.3)
				73.6 ± 7.6	50	14.6 ± 3.4			
Van Roeder et al. (5)	Retro	606	AS	80 (77–84)	282 (606)	13.0 (8.4–18.3)	ACD, HF hospitalization	12	94 (15.5)
Sabatino et al. (12)	Retro	100	AS	80.7 ± 5.3	36 (55)	NR	Cardiovascular mortality, HF hospitalization	31	35 (35)
				82 ± 5.4	16 (46)	NR			
Sonaglioni et al. (10)	Retro	186	AS	71.9 ± 12.7	115 (61.8)	24.9 ± 8.3	AVR, CV hospitalization, ACD	27.60 ± 22.80	63 (34)
Springhetti et al. (9)	Retro	467	AS	80.6 ± 8.2	237 (50.7)	20.0 ± 9.3	ACD, HF hospitalization	19.20 (12.50–24.40)	96 (21)
Stolz et al. (8)	Retro	1,888	AS	81.0 ± 7.8	1,052 (56)	16.5 ± 9.4	ACD	36	557 (29.5)
Tan et al. (26)	Prosp	173	AS	69 ± 11	95 (54.9)	27.2 (22.3–32.2)	ACD, HF hospitalization, NYHA ≥ III, ACS, syncope	32.40 (16.80–55.20)	66 (38)
Tan et al. (7)	Prosp	220	AR	49 (36–56)	175 (79.5)	27.05 (22.40–30.84)	ACD, HF hospitalization, AVR	12 (24.50–62.30)	46 (20.9)
Thellier et al. (6)	Retro	387	AS	76 (75–77)	181 (47)	24 (17–33)	ACD	57 (37–83)	158 (41)
Weber et al. (4)	Retro	150	AS	82 ± 8	63 (42)	21.96 ± 9.4	New onset of AF, HF hospitalization, ACD	5.7 (0.70–24.20)	37 (25)
Wedin et al. (3)	Prosp	227	AS	65.3 ± 9.2	87 (65.4)	22.2 ± 10.8	POAF, TIA or stroke	44.7	POAF64 (48.1); TIA/stroke21(16)
				71.2 ± 7.1	53 (56.4)	28.1 ± 9.4		52	POAF50(53.1); TIA/stroke5(5)

ACD, all cause death; AF, atrial fibrillation; AVR, aortic valve replacement; HF, heart failure; LVEF, left ventricular ejection fraction; LVEDVI, left ventricle end-diastolic volume index; MACE, major adverse cardiovascular events; NR, not reported; NYHA, New York Heart Association; PALS, peak atrial longitudinal strain; POAF, postoperative atrial fibrillation; TIA, transient ischemic attack.

### Relationship between PALS and events

**Figure 2:**
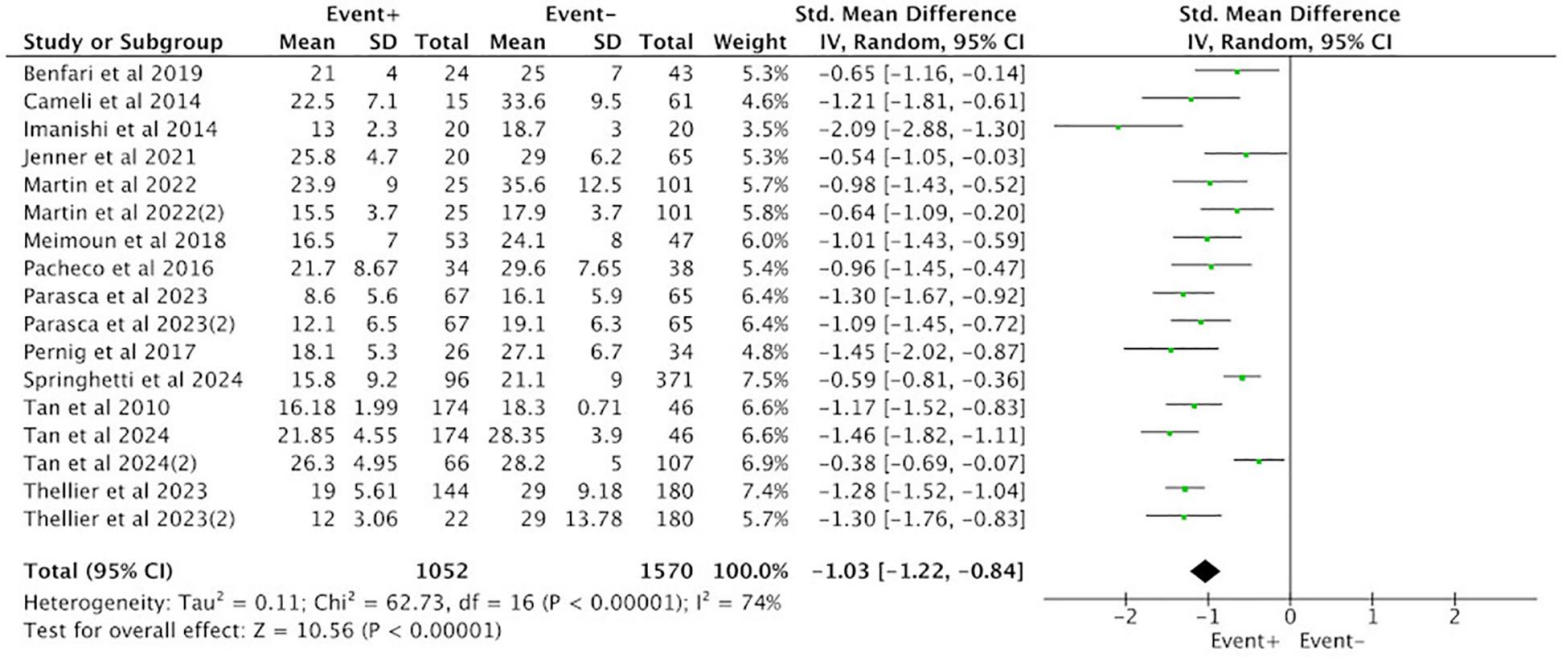
Difference in PALS between the positive group (EVENT+) and the negative group (EVENT−).

Thirteen studies reporting on PALS were included, involving a total of 2,622 patients, with 1,052 cases in the positive group (EVENT+) and 1,570 cases in the negative group (EVENT−). Heterogeneity analysis revealed that the included studies lacked good homogeneity (*p* < 0.05, *I*^2^ = 74%), so a random-effects model was used for analysis. The results showed that the difference in PALS between the positive group (EVENT+) and the negative group (EVENT−) reached statistical significance (SMD = −1.03, 95% CI [−1.22, −0.84], *p* < 0.05), indicating that there was a significant difference in PALS between the positive group (EVENT+) and the negative group (EVENT−) (Figure 2).

**Figure 3:**
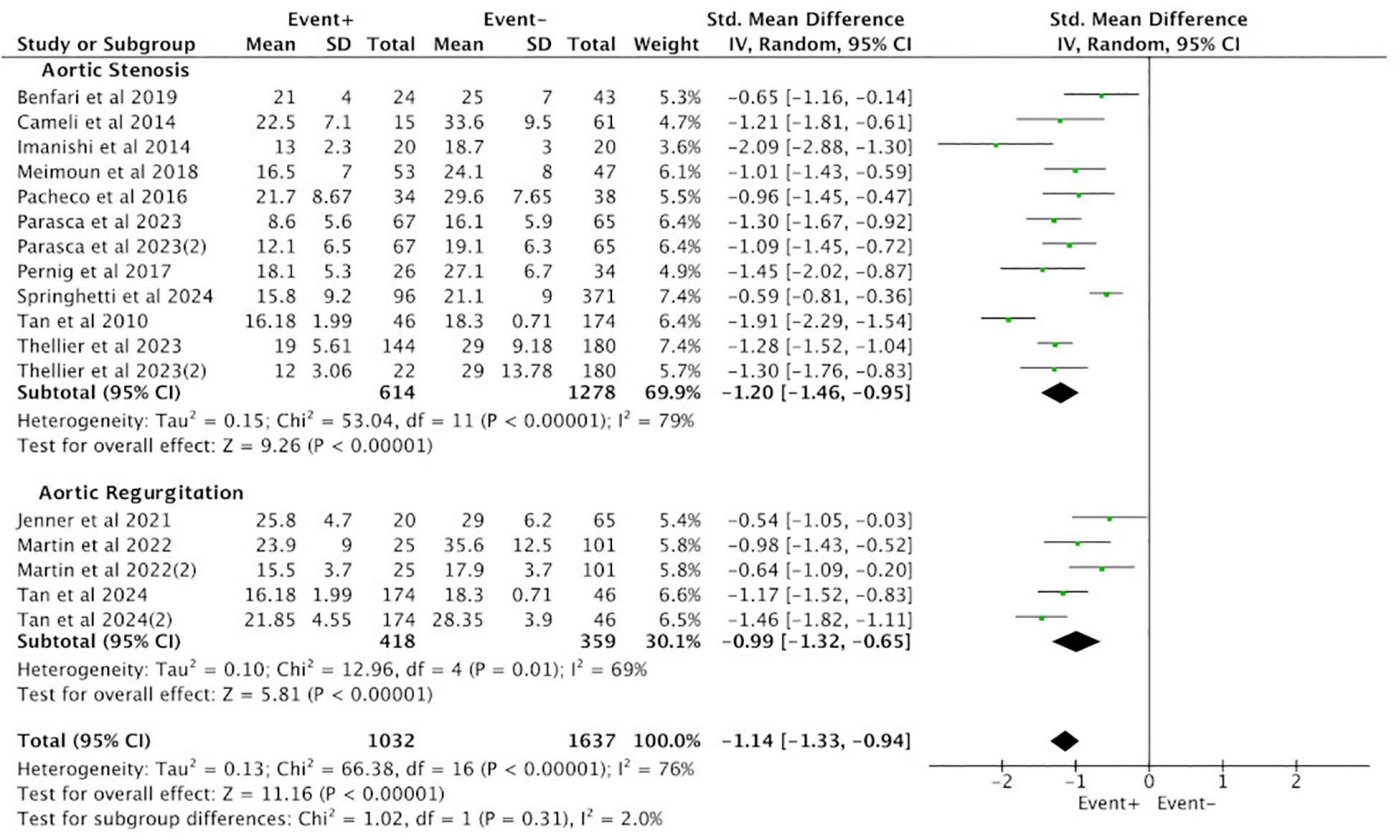
Subgroup analysis of difference in PALS between the positive group (EVENT+) and the negative group (EVENT−).

Subgroup analysis was conducted between the positive group (EVENT+) and the negative group (EVENT−) based on the type of aortic valve lesions, and the results were consistent with the overall findings. Among the 10 studies reporting PALS in the AS group, a total of 1,892 cases were included, with 614 cases in the positive group (EVENT+) and 1,278 cases in the negative group (EVENT−). Heterogeneity analysis revealed poor homogeneity among the included studies (*p* < 0.05, *I*^2^ = 79%), so a random-effects model was used for analysis. The results showed that the difference in PALS between the positive group (EVENT+) and the negative group (EVENT−) reached statistical significance (SMD = −1.20, 95% CI [−1.46, −0.95], *p* < 0.05), indicating a significant difference in PALS between the positive group (EVENT+) and the negative group (EVENT−) in the AS subgroup (Figure 3).

Three studies in the AR group reported PALS, involving a total of 777 cases, with 418 cases in the positive group (EVENT+) and 359 cases in the negative group (EVENT−). Heterogeneity analysis revealed poor homogeneity among the included studies (*p* < 0.05, *I*^2^ = 69%), so a random-effects model was used for analysis. The results showed that the difference in PALS between the positive group (EVENT+) and the negative group (EVENT−) reached statistical significance (SMD = −0.99, 95% CI [−1.32, −0.65], *p* < 0.05), indicating a significant difference in PALS between the positive group (EVENT+) and the negative group (EVENT−) in the AR subgroup (Figure 3).

### Meta-analysis of PALS on composite endpoint

**Figure 4:**
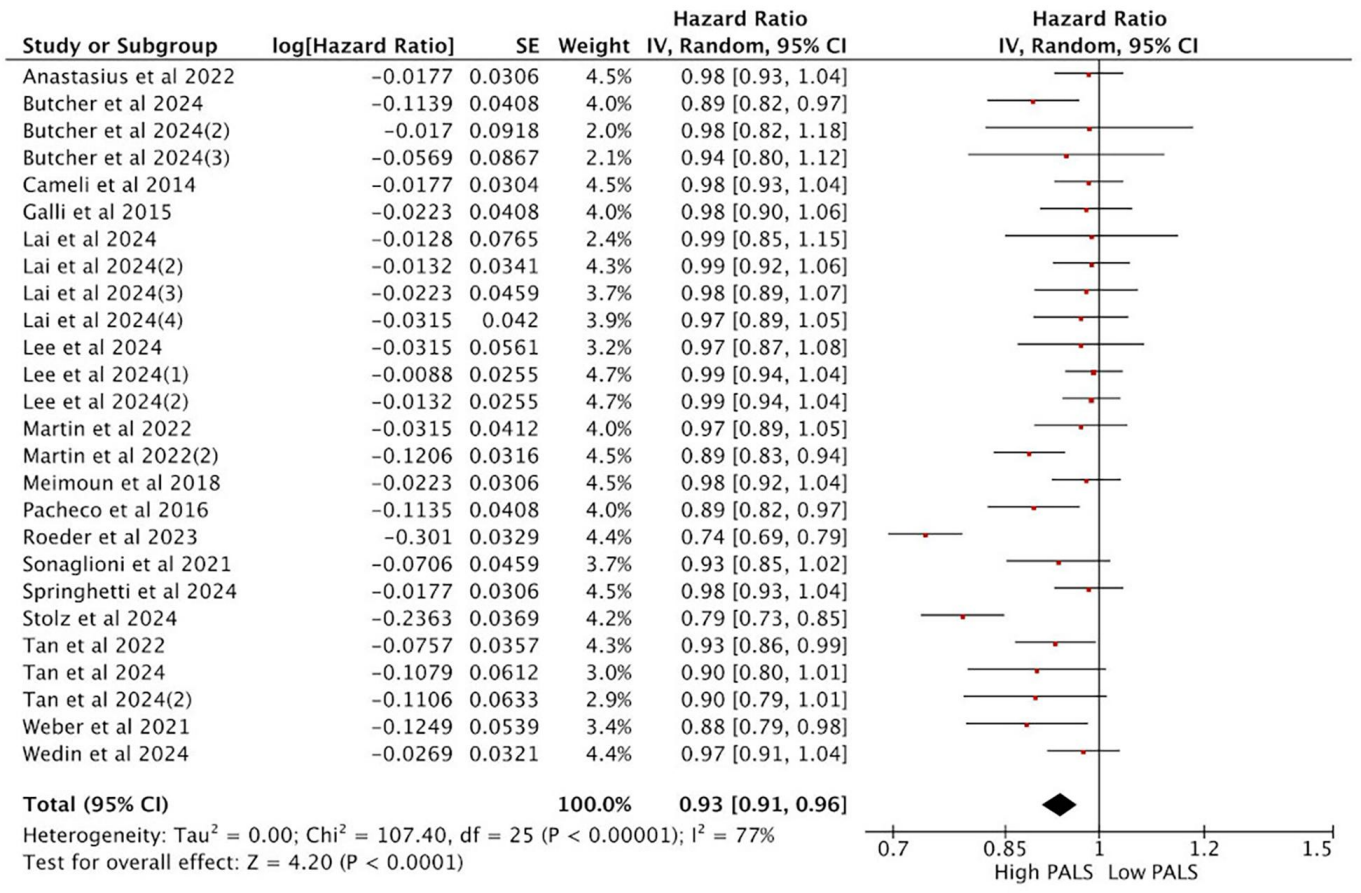
Meta-analysis of the relationship between PALS and composite endpoint.

A total of 17 studies reporting PALS were included in the analysis. Heterogeneity analysis revealed a lack of good homogeneity among the included studies (*p* < 0.05, *I*^2^ = 77%), so a random-effects model was used for analysis. The results indicated that, among the 17 studies reporting PALS, high PALS was associated with a statistically significant increased HR for the endpoint event compared with low PALS. Figure 4 illustrates the relationship between PALS and the incidence of endpoint events in a multivariate Cox regression model. Specifically, based on the adjusted HR from all 17 studies, each unit increase in PALS was associated with a 7% reduction in the risk of the primary endpoint event (HR = 0.93, 95% CI [0.91, 0.96], *p* < 0.001).

### Subgroup analysis of the relationship between PALS and composite endpoint

**Figure 5:**
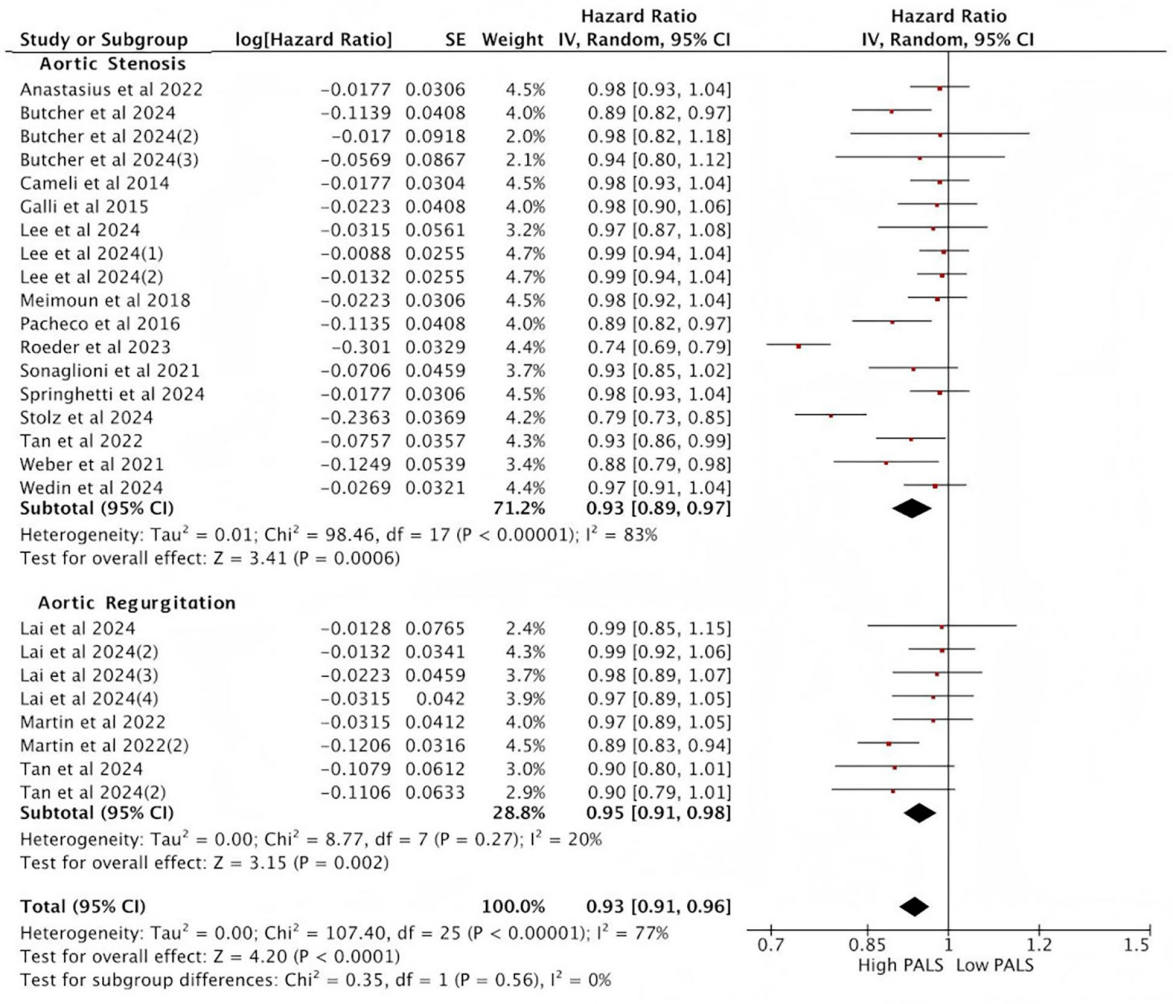
Subgroup analysis of the relationship between PALS and composite endpoint.

In subgroup analyses based on aortic valve lesion types, the pooled HR from the multi-variable Cox regression model was consistent with the results of the overall analysis. Among the 14 studies reporting PALS in AS, the pooled HR for predicting endpoint events was statistically significant between high PALS and low PALS. In the AS subgroup, each unit increase in PALS was associated with a 7% reduction in the risk of the primary endpoint (HR = 0.93, 95% CI [0.89, 0.97], *p* < 0.001) (Figure 5). In AR, 3 studies reported PALS, and the combined HR for high PALS compared with low PALS was statistically significant for predicting endpoint events. In the AR subgroup, each unit increase in PALS was associated with a 5% reduction in the risk of the primary endpoint (HR = 0.95, 95% CI [0.91, 0.98], *p* < 0.001) (Figure 5).

### The addition of PALS to baseline risk models

The addition of PALS to baseline risk models contributed to the increase of the C-statistic or the NRI (Table 2). Two studies consistently demonstrated that adding PALS variables to the baseline model significantly improved risk prediction for patients with AR. Specifically, the C-statistic increased from 0.78 to 0.80 and from 0.81 to 0.82, respectively, indicating that PALS enhances the ability to identify high-risk AR patients. In patients with AS, three studies reached similar conclusions. After incorporating PALS into the baseline model, the C-statistic rose significantly from 0.73 to 0.82 and from 0.77 to 0.79, respectively. A large-scale study (*n* = 923) further quantified the reclassification benefit using the NRI, yielding an NRI of 0.224 (95% CI: 0.07–0.38). This indicates that PALS correctly reclassified 22.4% of patients into more appropriate risk categories, with the confidence interval excluding zero, confirming both statistical significance and clinical relevance.

**Table 2:** The addition of PALS to baseline risk models.

Study	Subgroup	*N*	Baseline model	Baseline C-statistics	Baseline + PALS C-statistics	NRI (95% CI)
Jenner et al. (20)	AR	80	Age, Gender, LVEDVi, LVESVi, LVEF, LVGLS, Stroke work, DD grade, E/e′	0.78	0.80	NR
Lai et al. (19)	AR	352	Age, CCI, Gender, LVGLS	0.81	0.82	NR
Benfari et al. (25)	AS	67	Age, Gender, BSA, HT, DM	0.73	0.82	NR
Thellier et al. (6)	AS	387	Age, Gender, BSA, BMI, SBP, DBP, HT, DM, CAD, CCI	0.77	0.79	NR
Lee et al. (18)	AS	923	Age, Sex, HT, AF, history of HF, IHD, COPD, hemoglobin, AVR as a time-dependent covariate	NR	NR	0.224 (0.07–0.38)

AF, atrial fibrillation; AVR, aortic valve replacement; BMI, body mass index; BSA, body surface area; CAD, coronary artery disease; CCI, Charlson comorbidity index; COPD, chronic obstructive pulmonary disease; DM, diabetes mellitus; DBP, diastolic blood pressure; DD, diastolic dysfunction; HF, heart failure; HT, hypertension; IHD, ischemic heart disease; LVEDVi, left ventricular end diastolic volume index; LVESVi, left ventricular end-systolic volume index; LVEF, left ventricular ejection fraction; LVGLS, left ventricular global longitudinal strain; NRI, net reclassification index; NR, not reported; SBP, systolic blood pressure.

In summary, PALS delivers robust incremental prognostic value in both AR and AS cohorts, yet its magnitude and underlying mechanisms differ between the two groups. In AR, the gain conferred by PALS is consistent but modest, likely because the baseline model already incorporates sensitive parameters such as LVGLS. Conversely, in AS, PALS contributes more markedly, especially when the baseline model's discriminative performance is suboptimal. Furthermore, the NRI analysis substantiates PALS's pivotal role in refining individualized risk stratification for AS patients. Therefore, PALS is indispensable in aortic valve disease: it reliably enhances risk discrimination for AR, while for AS it not only significantly improves model discrimination but—via a validated NRI—also demonstrates powerful reclassification capacity, underscoring its necessity to overcome the limitations of traditional risk models.

### Sensitivity analysis

**Figure 6:**
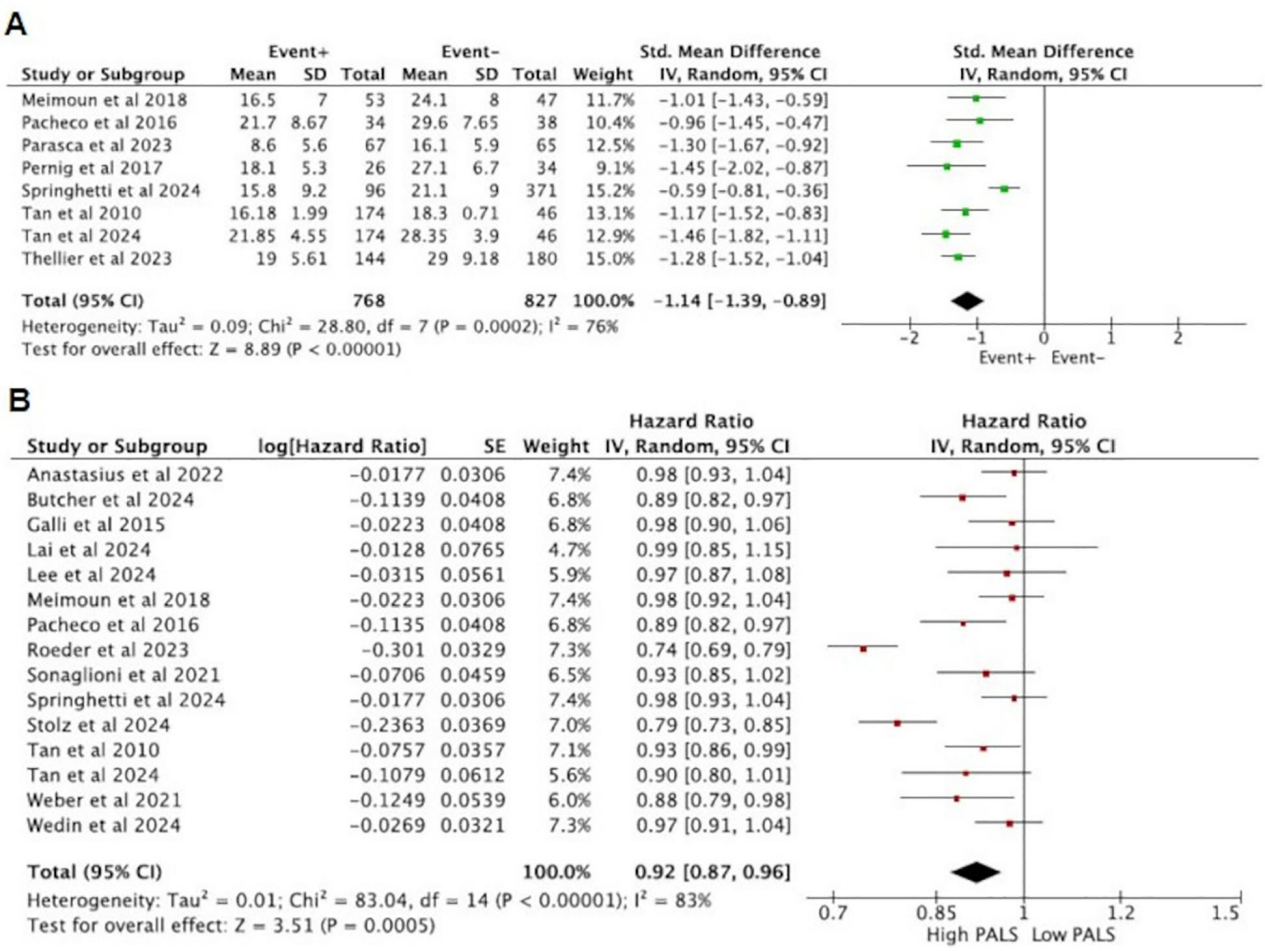
Sensitivity analysis results **(A)** and **(B)**.

Given that studies with small sample sizes tend to have lower reliability, this study excluded studies with single-group sample sizes less than 25 and reanalyzed the data. The results showed that the difference in PALS between the positive group (EVENT+) and the negative group (EVENT−) reached statistical significance (SMD = −1.14, 95% CI [−1.39, −0.89], *p* < 0.05), indicating that the PALS scores between the positive group (EVENT+) and the negative group (EVENT−) remained significantly different (Figure 6A). Additionally, the combined HR for predicting endpoint events between high PALS and low PALS was statistically significant. Specifically, for each unit increase in PALS, the risk of the primary endpoint event decreased by 7% (HR = 0.92, 95% CI [0.87, 0.96], *p* < 0.001) (Figure 6B). Therefore, the results of this study passed the sensitivity analysis.

### Meta-regression analysis

In this study, meta-regression analysis was further employed to identify and quantify the sources of heterogeneity. By constructing statistical models, we explored whether the differences in effect sizes among studies were driven by specific covariates. The selection of indicators primarily focused on three core dimensions of heterogeneity: methodological heterogeneity, population characteristic heterogeneity, and clinical intervention heterogeneity. Indicators such as PALS cutoff value, age, hypertension, LVEF, and endpoint events (death) were selected respectively. As shown in the results of the meta-regression analysis in Table 3, the difference in the definition of the mortality endpoint is a source of heterogeneity that has statistical significance for the effect size of all-cause death, while indicators such as PALS cutoff value, age, and hypertension do not have statistical significance. Based on the meta-regression results, it can be preliminarily concluded that the inconsistency in the definition and criteria for endpoint events among studies is one of the causes of heterogeneity. Additionally, the failure to identify significant effects of other factors may be related to the limited number of studies included and insufficient statistical power.

**Table 3:** Results of meta-regression analysis.

Sources	Coefficient	SE	*t*	*p* value
AGE	0.02	0.01	1.25	0.21
PALS-Cutoff	−0.19	0.48	−0.40	0.69
Death	−0.66	0.37	−1.77	0.08
Hypertension	−0.01	0.01	−1.20	0.23
LVEF	−0.02	0.02	−0.80	0.43
Constant	0.10	1.76	0.05	0.96

### Quality assessment

**Figure 7:**
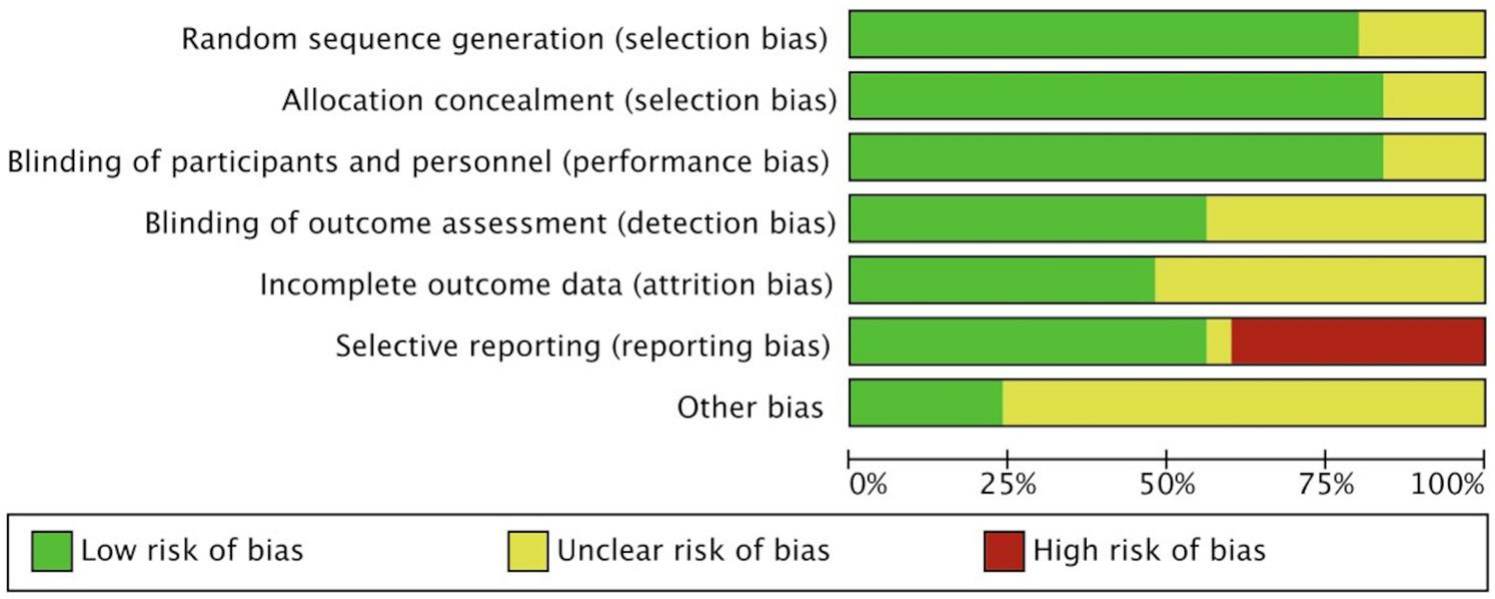
Cochrane risk of bias assessment tool for quality evaluation of included studies.

The Cochrane risk of bias assessment tool evaluated the quality of the included articles and found that most types of bias were low risk (green) in the trials, indicating that most studies were of good methodological quality, but there were some differences in certain aspects such as selection bias. All studies clearly defined their study subjects, patient groups, and outcomes (Figure 7, Supplementary Figure 1).

Despite the comprehensive assessment of the prognostic value of PALS in severe aortic valve disease through systematic review and meta-analysis in this study, there are still certain risks of bias and limitations in generalizability. According to the Cochrane Risk of Bias Assessment Tool (Figure 7), some studies had unclear or high risk of selection bias, mainly reflected in the incomplete reporting of patient inclusion criteria and baseline characteristics. Moreover, the majority of the included studies were single-center (20/25) and retrospective in design (15/25), which may introduce selection bias. In terms of geographical distribution, 21 studies were from European populations, with fewer studies from Asia and North America, limiting the generalizability of the results on a global scale. Table 4 summarizes the main limitations of this study and their potential impacts.

**Table 4:** Summary of limitations of the included studies.

Type of limitation	Specific manifestations	Potential impacts
Retrospective design	15 studies were retrospective cohort studies.	Selection bias may be introduced, affecting causal inference.
Single-center study	20 studies had a single-center design.	The representativeness of the sample and the external validity are limited.
Geographical bias	21 studies were based on European populations, with fewer studies from Asia and North America.	The findings may not be applicable to other ethnic groups and healthcare settings.
Selection bias	Patient enrollment process or baseline matching was not clearly reported in 12 studies.	The comparability between groups and the reliability of the results may be affected.
Variability in PALS measurement	Different ultrasound devices and software were used to measure PALS in the included studies.	Measurement heterogeneity

### Risk of bias

According to the funnel plots (Supplementary Figures S2–S5), the results of all analyses show that the variability of study effects increases with the increase in standard error, which is consistent with the greater variability and wider confidence intervals typically observed in studies with smaller sample sizes. Furthermore, all funnel plots exhibit a symmetrical distribution, suggesting that the likelihood of substantial bias in this study is low. Therefore, it is considered that the likelihood of reporting bias in this study is low.

### Quality in prognostic studies (QUIPS)

To more systematically and specifically assess the methodological quality of the observational prognostic studies included in this analysis, QUIPS tool was further employed for evaluating the risk of bias in prognostic studies in this study. The QUIPS framework evaluates studies across six key domains: study participation, study attrition, prognostic factor measurement, outcome measurement, confounding, and statistical analysis and reporting. This approach allows for a more precise identification of potential sources of bias in observational prognostic studies, thereby enhancing the rigor and comprehensiveness of the quality assessment in this study.

The results in Table 5 showed that all studies had a low risk in the measurement of prognostic factors, outcome assessment, and statistical analysis, which ensured the reliability of the core conclusions. However, there were significant limitations in study participation (11.54% medium risk), study attrition (34.62% medium risk), and confounding control (34.62% medium risk). These limitations were primarily due to the fact that most of the included studies had a single-center retrospective design, which may lead to selection bias and affect the generalizability of the results. This is consistent with the previous assessment results of the Cochrane tool and provides a more detailed analysis of the sources of bias.

**Table 5:** Quality assessment results of prognostic studies based on the QUIPS framework.

Bias	Low risk	Moderate risk	High risk
Study participation	23 (88.46%)	3 (11.54%)	0 (0.00%)
Study attrition	17 (65.38%)	9 (34.62%)	0 (0.00%)
Prognostic factor measurement	26 (100.00%)	0 (0.00%)	0 (0.00%)
Outcome measurement	26 (100.00%)	0 (0.00%)	0 (0.00%)
Study confounding	17 (65.38%)	9 (34.62%)	0 (0.00%)
Statistical analysis and reporting	26 (100.00%)	0 (0.00%)	0 (0.00%)

## Discussion

To the best of our knowledge, this meta-analysis is the first systematic collection and quantitative synthesis of reports on the prognostic value of PALS in significant aortic valve disease. An analysis of 25 studies involving 7,195 patients revealed the following findings: (1) PALS was lower in patients with endpoint events compared to those without endpoint events. (2) The risk of major endpoint events decreased with increasing PALS. PALS is an independent prognostic predictor in patients with severe aortic valve disease, and this holds true in both AS and AR patients. (3) PALS demonstrated robust incremental predictive value in both the AR and AS groups, yet its effect size and underlying mechanisms differed between the two cohorts. In summary, our findings emphasize the important clinical value of assessing LAS in patients with significant aortic valve disease.

AS primarily leads to increased LV pressure overload, resulting in concentric hypertrophy; AR, however, not only causes increased LV volume overload but also increased LV pressure overload, leading to LV dilation and eccentric remodeling (27). Both conditions may result in increased LV wall tension, particularly AR, where sustained high wall tension activates inflammatory responses in the myocardium, ultimately causing structural and functional damage to the heart muscle. Theoretically, the LA and LV are anatomically connected and function as LV filling regulators during different cardiac cycles to maintain cardiac output. Therefore, persistently increased LV filling pressure is transmitted to the LA via the atrioventricular connection. The LA must then contract more actively to resist the elevated LV filling pressure, which may lead to loss of atrial wall compliance and impaired LA contraction function (7). Recent studies have shown that LA strain impairment occurs earlier than LA dilation, particularly in PALS, which has garnered attention as a potential marker of LV diastolic dysfunction. Less load-dependent and more sensitive than conventional indices, it also emerges as an independent predictor of all-cause mortality in the general population, surpassing LVGLS in prognostic accuracy (17). However, in recent years, research has mostly used PALS as an indicator of LV diastolic dysfunction. But in theory, since PALS occurs during LV systole, when the LV contracts and pulls the LA towards the apex, PALS theoretically also reflects the longitudinal contraction ability of the LV. Moreover, PALS can also reflect the elasticity and stiffness of the LA myocardium. Atrial walls are thin and sensitive to pressure and volume changes. Additionally, the LA is connected to the pulmonary veins and serves as a blood reservoir. If its function is impaired, it may more directly lead to symptoms such as chest tightness and shortness of breath, which are associated with endpoint events. In this study, PALS was significantly lower among patients who reached the primary endpoint than among those who did not, thereby corroborating our earlier hypothesis.

Current guidelines recommend intervention for patients with symptomatic or LV systolic dysfunction (LVEF < 50%) and significant AS. For patients with symptomatic or asymptomatic significant AR, aortic valve surgery should be performed if the LV end-systolic diameter is >50 mm or the LV end-diastolic diameter is >65 mm (28). As can be seen, the current timing of intervention for severe active valve disease primarily depends on LV parameters, with less attention given to LA. However, these recommendations stemmed from studies with limited sample sizes and relatively outdated data. Recent evidence indicated that the extent and pattern of LV remodeling were influenced by age and sex: women and elderly patients with AR tended to maintain comparatively smaller LV dimensions, yet most experienced adverse cardiovascular events before reaching guideline-recommended LV size thresholds. In contrast, PALS showed a similar trajectory across age and sex groups; therefore, using PALS to inform operative timing may be superior to relying on LV parameters alone (29). In the future, LV criteria could be supplemented in AR patients with gender/age bias, or PALS parameters could be combined with LV parameters to determine the timing of surgery. This study employed C-statistic and NRI to evaluate the incremental prognostic value of PALS over traditional risk models. PALS conferred robust incremental predictive power in both AR and AS cohorts. Among AR patients, PALS consistently enhanced the discriminatory capacity of the risk model, whereas among AS patients it not only markedly improved model discrimination but also demonstrated reclassification efficacy as validated by the NRI, underscoring its necessity in addressing the limitations of conventional risk models. Consequently, integrating PALS into clinical risk stratification and surgical-timing decisions is likely to confer greater benefit to patients.

Regarding the value of LAS in aortic valve disease, there have been no previous meta-analyses evaluating the prognostic value of LAS in aortic valve disease. Only one qualitative systematic review, which included 18 studies involving 2,660 patients, was conducted, and it focused solely on AS and did not include AR (30). However, recent studies have suggested that LAS may also have prognostic value in AR. This meta-analysis included a total of 25 articles involving 7,195 patients, of which 4 studies reported only on AR, 20 reported only on AS, and 1 reported on both AS and AR. Compared with previous systematic reviews, the number of articles included and the number of patients involved have increased. Moreover, this study not only investigated the value of LAS in AS but also in AR, so the results of this study are widely representative and applicable to patients with severe aortic valve disease. As far as we know, this has not been reported before. Lacy et al. conducted a systematic review of 4 studies involving 822 patients and concluded that patients with AS who experienced adverse events had significantly lower PALS compared to those without events. This is consistent with our findings, but we included 10 studies involving 2,669 patients (10 for AS and 3 for AR). Compared with previous studies, our study has improved in the number of articles included, the number of patients, and the types of diseases. More importantly, in the research on prognosis, previous studies only systematically reviewed the value of PALS in the prognosis of AS without conducting a quantitative analysis. In contrast, we performed a meta-analysis of 17 articles (14 for AS and 3 for AR) and reached a quantitative conclusion. This has greater clinical value. Therefore, our study has taken a step forward based on the previous research.

After conducting subgroup analyses stratified by the type of aortic valve disease, it was observed that for every one-unit increase in PALS, the risk of major adverse cardiovascular events was reduced by 7% in patients with severe AS, compared to a 5% reduction in those with severe AR. This suggests that changes in PALS have a more pronounced impact on the prognosis of patients with AS. This difference is largely attributable to the distinct pathophysiological mechanisms underlying these two conditions. AS mainly leads to increased LV pressure load, while AR can lead to increased LV volume load and pressure load, which in turn activates different intracellular signaling pathways and leads to different types of cardiomyocyte hypertrophy and fibrosis patterns (28). Also, the type of LV hypertrophy presented by the patients with AR, with a chronic development of eccentric remodelling, probably better accommodates the increase of cavity filling pressures (31). At molecular level, Holst et al. conducted proteomic analyses on patients with severe isolated AS and AR and found that the LV myocardial proteome differed between AR and AS patients. AS was associated with higher levels of extracellular region proteins related to hematopoiesis/angiogenesis and tissue healing. In contrast, AR was associated with higher levels of intracellular region proteins related to energy production and cellular metabolism, which may indicate a compensatory “high metabolic intracellular myocardial state” to meet the increased energy demands (32). This could be the reason why patients with AR have a stronger compensatory capacity compared to those with AS. However, due to the limited number of articles on AR included in this study, the conclusions regarding the prognostic value of PALS in severe AR are only exploratory. Further research is needed in the future to validate our hypotheses.

In sensitivity analyses, we excluded small-sample studies and adjusted grouping criteria, yet the findings remained fully consistent. PALS retained prognostic value in significant aortic valve disease and served as an effective prognostic indicator, providing clinicians with new reference criteria.

## Limitation

This study is the first to comprehensively evaluate the prognostic value of PALS in significant aortic valve disease through systematic review and meta-analysis. However, this study also has certain limitations. First, the heterogeneity of the included studies. Meta-regression analysis was further employed to identify and quantify the sources of heterogeneity in this study. Meanwhile, subgroup analyses and sensitivity analyses were performed to analyze and eliminate the marked heterogeneity observed. Although heterogeneity still existed, the prognostic significance of PALS remained robust, indicating its broad applicability across diverse aortic valve disease populations. Currently, there are still certain challenges in the standardization of PALS cut-off points. Secondly, the number of studies on AR included in this study is limited, which means that the findings regarding the prognostic value of PALS in AR are only exploratory. This is also due to the fact that the application of PALS in AR has not yet been fully recognized, which indirectly reflects the value of this study. Finally, due to the limited number of included articles and insufficient data, it was not possible to determine a PALS cutoff value for predicting the prognosis of severe aortic valve disease. At present, there is no validated PALS threshold value available for clinical practice, and existing guidelines lack LA parameters for judging the timing of intervention. The possible reason is that the adoption of PALS in clinical practice will face many practical barriers, such as the need for training and the variability of software. At present, it is possible to consider combining PALS with LV parameters for risk stratification of aortic valve disease and selection of surgical timing. More large-sample studies are needed in the future to further investigate this area.

## Conclusion

PALS is an independent predictor of adverse cardiovascular events in patients with significant aortic valve disease. PALS has certain value in the prognosis of significant aortic valve disease and can provide new reference indicators for clinicians.

## Data Availability

The raw data supporting the conclusions of this article will be made available by the authors, without undue reservation.
