# Correction: Prognostic value of left atrial strain in significant aortic valve disease: a systematic review and meta-analysis

**DOI:** 10.3389/fcvm.2026.1787377

**Published:** 2026-02-10

**Authors:** Na Chen, Wenhui Gu, Jun Wu

**Affiliations:** 1 Department of Echocardiography, The Second Affiliated Hospital of Dalian Medical University, Dalian, China; 2 Department of Echocardiography, The First Affiliated Hospital of Dalian Medical University, Dalian, China

**Keywords:** aortic regurgitation, aortic stenosis, left atrial strain, meta-analysis, peak left atrial longitudinal strain, systematic review

Affiliation Department of Echocardiography, The Second Affiliated Hospital of Dalian Medical University, Dalian, China was omitted from the paper. This affiliation has now been added for author Na Chen.

In the published article, the ranking of the affiliations was listed incorrectly. The correct affiliation ranking reads:

Na Chen1,2, Wenhui Gu1 and Jun Wu1*

The original version of this article has been updated.

