# Supplementary material for: Prognostic value of left atrial strain in significant aortic valve disease: a systematic review and meta-analysis
Source: Front Cardiovasc Med. 2025 Sep 16;12:1667871. doi: 10.3389/fcvm.2025.1667871 (PMC12482921; doi:10.3389/fcvm.2025.1667871)
Supplement: Supplementary file 1 [file Datasheet1.pdf]

Supplemental material

Supplemental table S1. Complete search strategy for each database

Database	Search strategy	Articles searched
PubMed	("atrial function, left"[MeSH Terms] OR ("atrial deformation"[Title/Abstract] OR "atrial function"[Title/Abstract] OR "atrial functions"[Title/Abstract] OR "atrial longitudinal strain"[Title/Abstract] OR "atrial strain"[Title/Abstract] OR "left atrial function*"[Title/Abstract] OR "left atrium function"[Title/Abstract])) AND ("aortic valve Insufficiency"[MeSH Terms] OR ("aortic valve stenosis"[MeSH Terms] OR ("aortic"[All Fields] AND "valve"[All Fields] AND "stenosis"[All Fields]) OR "aortic valve stenosis"[All Fields]) OR ("aorta insufficiency"[Title/Abstract] OR "aorta regurgitation"[Title/Abstract] OR "aortic incompetence"[Title/Abstract] OR "aortic insufficiency"[Title/Abstract] OR "aortic regurgitation"[Title/Abstract] OR "aortic stenosis"[Title/Abstract] OR "aortic valve incompetence"[Title/Abstract] OR "aortic valve Insufficiency"[Title/Abstract] OR "aortic valve regurgitation"[Title/Abstract] OR "aortic valve stenos*"[Title/Abstract] OR "aortic valvular stenos*"[Title/Abstract] OR "stenosed aortic valve*"[Title/Abstract] OR "stenotic aortic valve*"[Title/Abstract] OR "valvular aortic stenos*"[Title/Abstract]))	263
Cochrane	1 :((atrial functions, left) OR ('left atrial function*' OR 'atrial function' OR 'atrial strain' OR 'atrial deformation 'OR 'atrial longitudinal strain'):ti,kw,ab)	138
	2: MeSH descriptor: [Aortic Valve Stenosis] explode all trees	
	3: MeSH descriptor: [Aortic Valve Insufficiency] explode all trees	
	4: (aorta insufficiency' OR 'aorta regurgitation' OR 'aorta semilunar valve incompetence' OR 'aorta valve incompetence' OR 'aorta valve insufficiency' OR 'aorta valve regurgitation' OR 'aorta valve stenosis' OR 'aortic incompetence' OR 'aortic insufficiency' OR 'aortic regurgitation' OR 'aortic stenosis' OR 'aortic valve incompetence' OR 'aortic valve Insufficiency' OR 'aortic valve regurgitation' OR 'aortic valve stenos*' OR 'aortic valvular stenos*' OR 'stenosed aortic valve*' OR 'stenotic aortic valve*' OR 'valvular aortic stenos*'):ti,kw,ab	
	5: 2 OR 3 OR 4	
	6: 1 AND 5	
Web of science	TS=(aortic valve Insufficiency OR aortic valve stenosis OR aorta insufficiency OR aorta regurgitation OR aorta semilunar valve incompetence OR aorta valve incompetence OR aorta valve insufficiency OR aorta valve regurgitation OR aorta valve stenosis OR aortic incompetence OR aortic insufficiency OR aortic regurgitation OR aortic stenosis OR aortic valve incompetence OR aortic valve Insufficiency OR aortic valve regurgitation OR aortic valve stenos* OR aortic valvular stenos* OR stenosed aortic valve* OR stenotic aortic valve* OR valvular aortic stenos*) AND TS= (atrial function, left OR atrial deformation OR atrial function OR atrial functions OR atrial longitudinal strain OR atrial strain OR left atrial function* OR left atrium function)	2002
Embase	('left atrial function'/exp OR ('atrial deformation ' OR 'atrial function' OR 'atrial functions' OR 'atrial longitudinal strain' OR 'atrial strain' OR 'left atrial function*' OR 'left atrium function'):ti,ab,kw) AND ('aortic valve stenosis'/exp OR 'aortic regurgitation'/exp OR 'aorta insufficiency':ti,ab,kw OR 'aorta regurgitation':ti,ab,kw OR 'aorta semilunar valve incompetence':ti,ab,kw OR 'aorta valve incompetence':ti,ab,kw OR 'aorta valve insufficiency':ti,ab,kw OR 'aorta valve regurgitation':ti,ab,kw OR 'aorta valve stenosis':ti,ab,kw OR 'aortic incompetence':ti,ab,kw OR 'aortic insufficiency':ti,ab,kw OR 'aortic regurgitation':ti,ab,kw OR 'aortic stenosis':ti,ab,kw OR 'aortic valve incompetence':ti,ab,kw OR 'aortic valve insufficiency':ti,ab,kw OR 'aortic valve regurgitation':ti,ab,kw OR 'aortic valve stenos*':ti,ab,kw OR 'aortic valvular stenos*':ti,ab,kw OR 'stenosed aortic valve*':ti,ab,kw OR 'stenotic aortic valve*':ti,ab,kw OR 'valvular aortic stenos*':ti,ab,kw)	311

Ovid	(Atrial Function/ or (atrial deformation or atrial function or atrial functions or atrial longitudinal strain or atrial strain or left atrial function* or left atrium function).ab,ti,kw.) and (Aortic Valve Insufficiency/ or Aortic Valve Stenosis/ or (aorta insufficiency or aorta regurgitation or aorta semilunar valve incompetence or aorta valve incompetence or aorta valve insufficiency or aorta valve regurgitation or aorta valve stenosis or aortic incompetence or aortic insufficiency or aortic regurgitation or aortic stenosis or aortic valve incompetence or aortic valve Insufficiency or aortic valve regurgitation or aortic valve stenos* or aortic valvular stenos* or stenosed aortic valve*).ab,ti,kw.)	87
CNKI	(Aortic valve stenosis + aortic valve insufficiency) OR TKA = (aortic stenosis + aortic regurgitation + aortic valve insufficiency + cardiac aortic valve insufficiency) AND (SU = (atrial function, left) OR TKA = (left atrial function + left atrial strain + left atrial longitudinal strain))	89

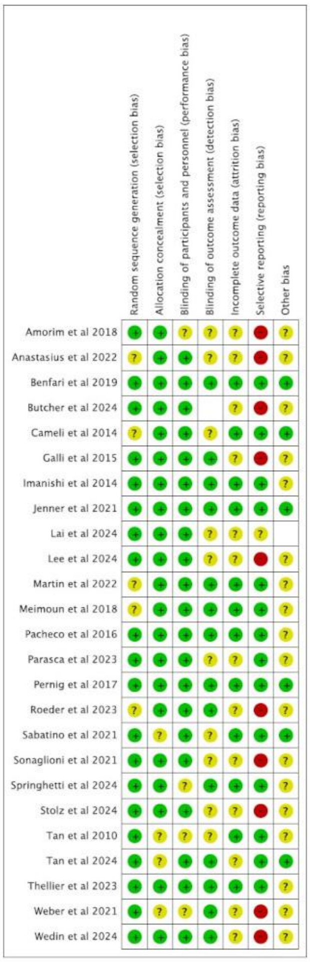

Supplemental figure S1 Specific quality evaluation of the included studies.

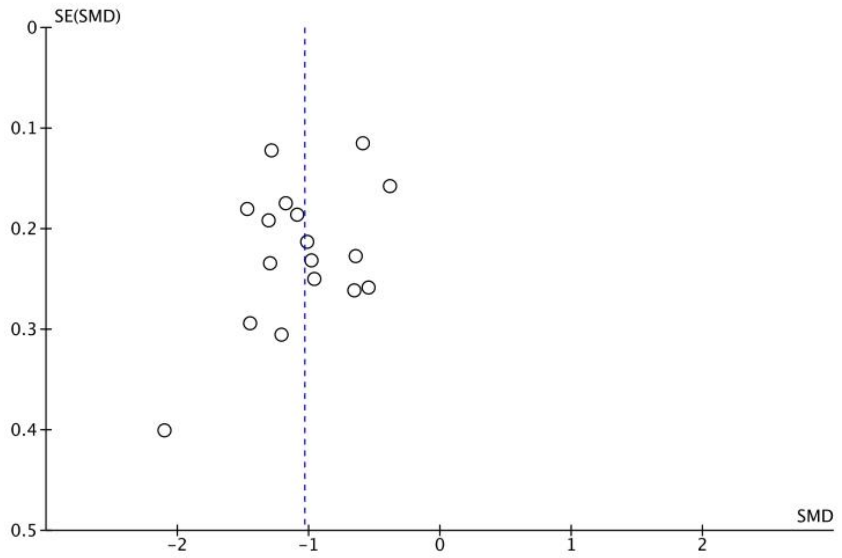

Supplemental figure S2 Funnel plot of PALS in EVENT (+) and EVENT (-) groups

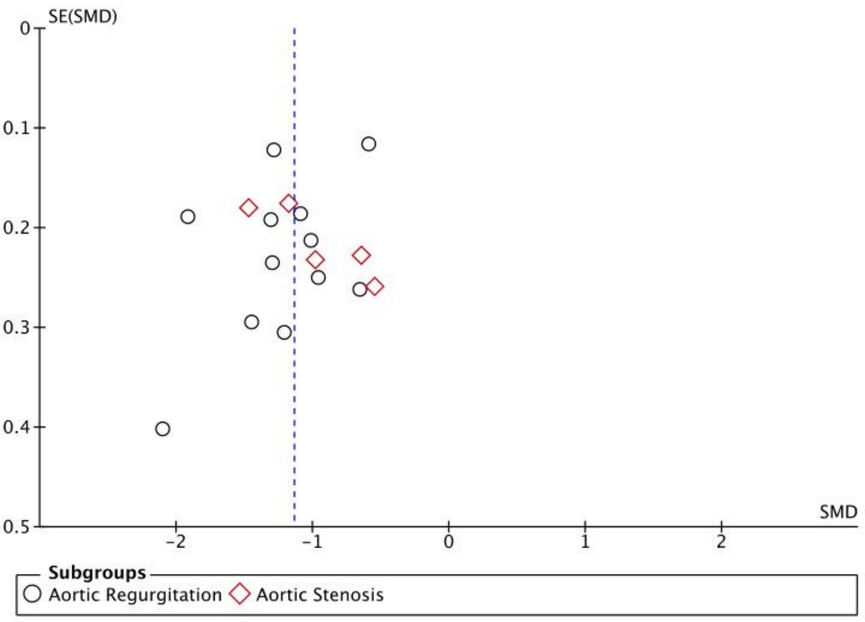

Supplemental figure S3 Funnel plot of subgroup analysis of PALS in AS and AR subgroups

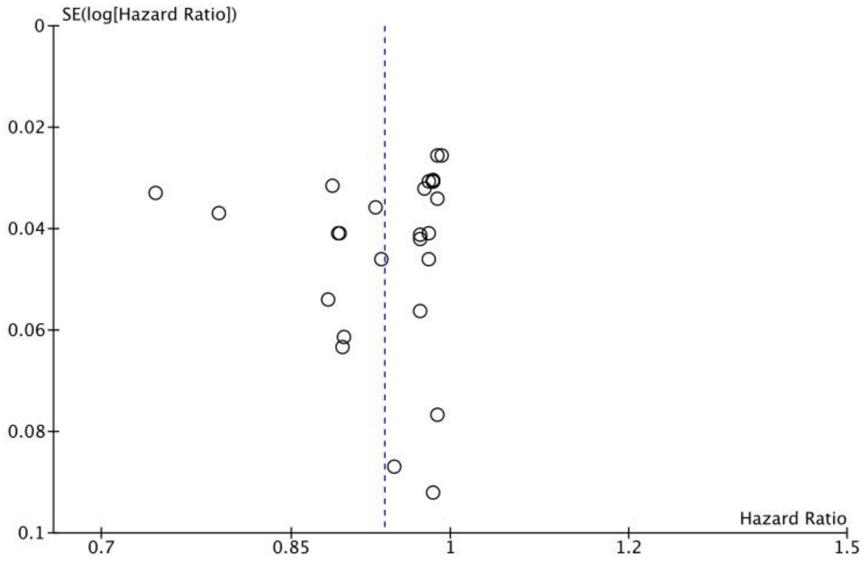

Supplemental figure S4 Funnel plot of the relationship between PALS and endpoint

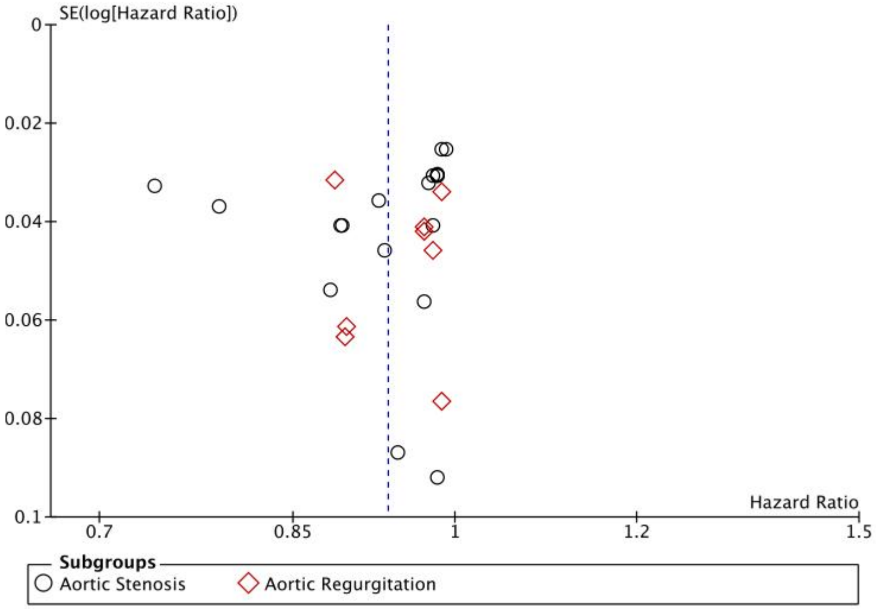

Supplemental figure S5 Funnel plot of subgroup analysis of the relationship between PALS and endpoint
